# Synthesis of Structure-Adjustable R-Au/Pt-CdS Nanohybrids with Strong Plasmon Coupling and Improved Photothermal Conversion Performance

**DOI:** 10.3390/nano14100838

**Published:** 2024-05-09

**Authors:** Hangyu Yan, Guowei Li, Fengyuan Zhang, Jingsong Liu, Mengdai Luoshan

**Affiliations:** School of Science, Hubei University of Technology, Wuhan 430068, China; yanhangyu@hbut.edu.cn (H.Y.); liguowei@hbut.edu.cn (G.L.); zhangfengyuan@hbut.edu.cn (F.Z.); liujingsong@hbut.edu.cn (J.L.)

**Keywords:** R-Au/Pt-CdS nanohybrids, nanostructure, surface plasmon resonance, photothermal conversion

## Abstract

Noble metal nanomaterials with a localized surface plasmon resonance effect exhibit outstanding advantages in areas such as photothermal therapy and photocatalysis. As a unique plasmonic metal nanostructure, gold nanobipyramids have been attracting much interest due to their strong specific local electric field intensity, large optical cross sections, and high refractive index sensitivity. In this study, we propose a novel three-component hetero-nanostructure composed of rough gold nanobipyramids (R-Au NBPs), Pt, and CdS. Initially, purified gold nanobipyramids are regrown to form R-Au NBPs that have a certain degree of roughness. These R-Au NBP substrates with a rough surface provide more hotspots and strengthen the intensity of localized electric fields. Subsequently, Pt and CdS nanoparticles are selectively deposited onto the surface of R-Au NBPs. Pt nanoparticles can provide more active sites. Each component of this hetero-nanostructure directly contacts others, creating multiple electron transfer channels. This novel design allows for tunable localized plasmon resonance wavelengths ranging from the visible to near-infrared regions. These factors contribute to the final superior photothermal conversion performance of the R-Au/Pt-CdS nanohybrids. Under the irradiation of near-infrared light (1064 nm), the photothermal conversion efficiency of R-Au/Pt-CdS reached 38.88%, which is 4.49, 1.5, and 1.22 times higher than that of Au NBPs, R-Au NBPs, and R-Au NBPs/Pt, respectively.

## 1. Introduction

The conversion of solar energy into heat by photothermal agents has received much attention in recent years. Many significant breakthroughs have been made in photocatalysis [1], photothermal therapy [2], and near-infrared (NIR) photothermal conversion [3]. In general, NIR is often divided into three segments: the first near-infrared bio-window (NIR-I, 750–950 nm), the second near-infrared bio-window (NIR-II, 1000–1700 nm), and the third near-infrared bio-window (NIR-III, 1450–1870 nm) [4,5]. In the NIR-I range, biological tissues absorb less light, allowing for relatively good light penetration. It is commonly used for non-invasive bio-imaging and therapy [6], while NIR-II has longer wavelengths, providing stronger penetration into biological tissues. Additionally, NIR-II has less light scattering and better biocompatibility. These properties enable NIR-II-absorbing photothermal conversion agents to generate heat at greater tissue depths, result in facilitating localized treatments [7,8,9,10].

In recent years, numerous NIR-II photothermal agents have been developed for efficient heat production, broadly categorized into monometallic plasmonic nanoparticles [11], bimetallic plasmonic nanoparticles [12], and metal-semiconductor nanostructures [13]. In particular, metal with a plasmon resonance effect exhibits an excellent ability to absorb specific wavelengths, leading to more efficient conversion of light energy into heat energy. Gold and silver nanoparticles, with their localized surface plasmon resonance effect, present good photothermal conversion efficiency owing to their tunability of incident light and enhanced localized electric field intensity [14,15,16,17,18]. For instance, Ding and coworkers prepared gold rods with adjustable surface roughness by depositing PbS, and the maximum photothermal efficiency reached 66.8% [19]. Bi and colleagues developed spiked gold nanoparticles and achieved a photothermal conversion efficiency of 78.8% under 980 nm light irradiation [20]. Chen and colleagues obtained bimetallic Au-Ag nanoparticles by modulating the molar ratio of silver-to-gold precursors and measured a photothermal efficiency of 41.37% [21]. Compared with Au and Ag, Pt with the lowest Fermi level exhibits more active sites and better catalytic properties, along with unique photothermal conversion properties. The combination of Au or Ag with Pt can effectively enhance the photothermal conversion efficiency and photocatalytic activity [22,23,24,25]. Duan et al. created a dumbbell-like structure by depositing Pt on the tip of Au rods. They reported its photothermal conversion efficiency at 78.76%, which was substantially higher than the efficiency of pure Au rods [26]. However, nanostructures composed of plasmonic metals still have limitations in photothermal conversion application due to restricted wavelength range, electron-hole transport efficiency, and size effects [27,28]. It is well known that the optical properties of plasmonic metal-based nanostructures are highly dependent on their morphology and components. Significant attention in photothermal conversion applications is devoted to the construction of multi-component nanostructures via metal and semiconductors [29,30,31]. Among them, CdS can efficiently generate photogenerated carriers and possesses superior chemical and thermal stability, creating favorable conditions for further photothermal conversion processes.

Herein, a specific hetero-nanostructure with three components is synthesized via a controllable wet chemistry method. Initially, the R-Au NBPs with a certain rough surface are prepared by varying the volume of the added Au NBPs. Subsequently, we controlled the position of surfactant molecules on R-Au NBPs by using the curvature effect [32] and facilitated the selective deposition speed of Pt nanoparticles. Finally, CdS is grown onto the remaining exposed surface of R-Au NBPs/Pt. This design ensures direct contact between each component, creating multiple efficient transfer pathways for the carrier [25]. The extinction spectra of these nanostructures demonstrate that shifts across the long-wave near-infrared region exhibit high tunability during the growth process. The photothermal conversion performance of these nanostructures with varying compositions is systematically evaluated. Under 1064 nm laser irradiation, the R-Au/Pt-CdS nanohybrids present superior photothermal conversion performance when compared to that of pure Au NBPs, R-Au NBPs, and R-Au NBPs/Pt under the same mass concentration. The enhancement of photothermal conversion efficiency can be attributed to the enhanced localized electric field intensity and light harvesting ability of R-Au NBPs with a rough surface. Meanwhile, Pt nanoparticles act as plasma damping units and can provide many active sites. The decorated CdS nanoparticles also benefit from constructing electron transfer channels, resulting in enhancing the final photothermal conversion efficiency.

## 2. Materials and Methods

### 2.1. Materials

Tetrachloroauric acid tetrahydrate (HAuCl_4_·4H_2_O), sodium citrate tribasic dihydrate (TSC, 99.0%), silver nitrate (AgNO_3_, 99.0%), hydrochloric acid (HCl, 36.0−38.0 wt%), L-ascorbic acid (AA, 99.0%), ammonia solution (NH_3_H_2_O, 25.0−28.0 wt%), hydrogen peroxide (H_2_O_2_, 30.0 wt%), hexamethylenetetramine (HMT, 99.5%), thioacetamide (TAA, 99%), and cadmium acetate (CH_3_COO)_3_Cd were purchased from Sinopharm Chemical Reagent Co. Ltd. (Shanghai, China). Sodium borohydride (NaBH_4_, 99%), hexadecyltrimethylammonium chloride (CTAC, 97%), hexadecyltrimethylammonium bromide (CTAB, 99.0%), sodium iodide (NaI, 99.5%), and potassium chloroplatinite (K_2_PtCl_6_, 99.5%) were purchased from Aladdin Reagent. The chemicals were not further purified and were used straight from the original packaging. Deionized (DI) water with a resistivity of 16.8 Ω·cm was used throughout the experiments.

### 2.2. Synthesis of R-Au NBPs

Au NBPs were synthesized and purified by using a seed-mediated approach as described in previous work [33,34]. After that, the purified Au NBPs (16 mL) were centrifuged at 6000 rpm for 10 min, and the precipitate was re-dispersed in DI water (2 mL). CTAB (0.1 M, 25 mL), HAuCl_4_ (25 mM, 0.25 mL), and AgNO_3_ (0.1 M, 75 μL) were added sequentially to a 50 mL centrifuge tube, then AA (0.1 M, 137 µL) was added to the mixture under vigorous stirring. Finally, the above Au NBP solution (0.5~1.75 mL) was added and allowed to preserve at room temperature for 12 h. The final product was redispersed into the CTAC solution after centrifugation at 6000 rpm for 10 min.

### 2.3. Synthesis of R-Au NBPs/Pt Nanostructures

In a typical synthesis, NaI (10 mM, 100 μL) was added into the CTAB solution (50 mM, 20 mL). Then, R-Au NBPs (5 mL), AgNO_3_ (5 mM, 30 μL), AA (0.1 M, 480 μL), and K_2_PtCl_6_ (1 mM, 75 μL) were added sequentially to the mixed solution, and then kept in an oven at 70 °C for 4 h. Finally, the product was centrifuged, washed, and redispersed into DI water for subsequent use.

### 2.4. Synthesis of R-Au/Pt-CdS Nanostructures

CTAB (0.1 M, 1 mL), AA (0.1 M, 1 mL), HTM (0.1 M, 1 mL), TAA (10 mM, 7 μL), and cadmium acetate (10 mM, 5 μL) were added sequentially to the prepared R-Au NBPs/Pt nanostructures (5 mL). The mixed solution was kept in oven at 80 °C for 8 h before being centrifuged at 6000 rpm for 10 min and redispersed into water.

### 2.5. Photothermal Conversion Efficiency Measurements

In this experiment, 1 mL of the sample was placed in a cuvette and irradiated by a 1064 nm laser beam with a power density of 1 W/cm^2^. Temperatures were recorded using a FORTRIC225 IR thermal imager (Shanghai Thermal Image Electro-Mechanical Technology Co., Ltd., Shanghai, China).

### 2.6. Sample Characterization

SEM observations were performed with a Zeiss Gemini scanning electron microscope 500 (Zeiss, Oberkochen, Germany) operated at an accelerating voltage of 25.0 kV. TEM images were obtained by operating a JEM-2100 (JEOL, Tokyo, Japan) transmission electron microscope at 200 KV. UV-vis spectra were recorded by spectrophotometric measurements on a (Hitachi High-Tech Corporation, Schaumburg, IL, USA). EDS analysis was conducted on an energy-dispersive X-ray spectrometer installed in the TEM. The X-ray photoelectron spectra were measured by a ESCALAB250Xi (Thermo Fisher Scientific, Waltham, MA, USA) X-ray photoelectron spectrometer.

## 3. Results and Discussion

### 3.1. Structural Morphologies and Optical Properties of R-Au NBPs

**Figure 1:**
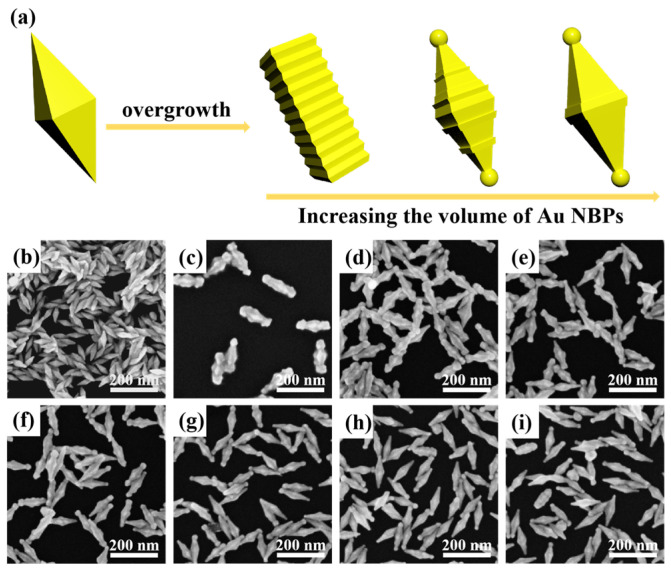
(**a**) Schematic illustration of the synthetic process of R-Au NBPs. SEM image of pure Au NBPs (**b**). SEM images of R-Au NBPs with different volumes of the added pure Au NBPs. The added volume of Au NBP solution was 0.5 mL (**c**), 0.75 mL (**d**), 1 mL (**e**), 1.25 mL (**f**), 1.5 mL (**g**), 1.75 mL (**h**), and 2 mL (**i**).

We successfully synthesized R-Au NBPs with varying degrees of roughness via a regrowth method, which is schematically shown in Figure 1a. Briefly, the purified Au nanobipyramids (Au NBPs) with highly uniform morphologies were obtained. In Figure 1b, the average longitudinal length (tip-to-tip) and average transverse width (equatorial length) of these synthesized Au NBPs are 81.23 ± 3 nm and 28.26 ± 3 nm, respectively. Then, using these Au NBPs as seeds, we introduced them into a growth solution containing HAuCl_4_, AgNO_3_, and ascorbic acid (AA), which guaranteed the regrowth of Au onto the surface of Au NBPs. In this case, AgNO_3_ acted as a surfactant and slowed down the reduction speed of HAuCl_4_, causing the anisotropic growth of Au onto the surface of the original Au nanobipyramids [35]. At first, the volume of Au NBPs was 0.5 mL, and the growth solution was relatively excessive, leading to the overgrowth of Au onto the surface of Au NBPs, ultimately forming rough Au nanorods, as shown in Figure 1c. The average longitudinal and transverse lengths of these rough Au nanorods were 147.06 ± 3 nm and 60.63 ± 3 nm, respectively. Following the increase in the volume of the introduced Au NBPs, Au gradually grew on the side surfaces and ends of the Au NBPs, and R-Au NBPs with varying degrees of roughness were obtained. As shown in Figure 1d, when the volume of the added Au NBPs was 0.75 mL, the R-AuNBPs underwent a transition from a rod-shaped morphology to a biconical shape, resulting in R-Au NBPs with average longitudinal and transverse lengths of 134.9 ± 3 nm and 45.9 ± 3 nm. However, with the further increase in Au NBP solution, the growth solution was gradually not enough. Due to the curvature effect, a small amount of Au tended to only grow at the ends and near the equator of the Au NBPs, forming a dumbbell-like structure (Figure 1e–g). The average longitudinal lengths reduced from 132.12 ± 3 nm to 128.36 ± 3 nm, and the transverse lengths reduced from 45.35 ± 3 nm to 43.43 ± 3 nm. When the added Au NBP solution was overdosed, the growth of Au at the ends and the equator of the Au NBPs gradually decreased; in contrast, the surface of R-Au NBPs became smooth (Figure 1h,i). The average longitudinal length and average transverse length decreased to 123.67 ± 3 nm and 42.34 ± 3 nm (Figure 1i).

**Figure 2:**
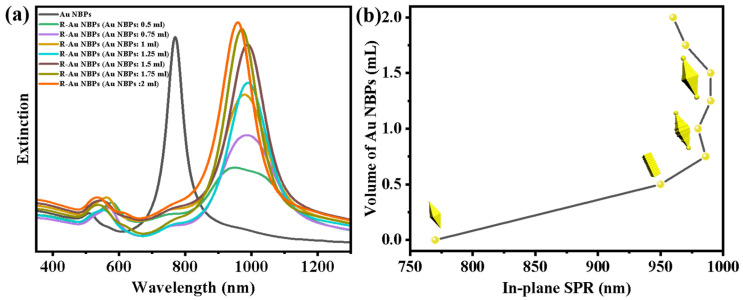
(**a**) Experimental extinction spectra of Au NBPs and R-Au NBPs with different Au NBP solution volumes. (**b**) The relationship plots between the added volume of Au NBPs and in-plane plasmon resonance peak.

The evolution of extinction spectra along with the changing degree of roughness is shown in Figure 2a. Equal amounts of all the samples are measured under similar conditions. The initial Au NBPs have a sharp main peak located at 770 nm and a weaker peak around 510 nm, which are attributed to transverse and longitudinal plasmon resonance modes, respectively. After the regrowth of Au, the main peak of R-Au NBPs broadened and gradually red-shifted to around 950 nm, due to the increase in size and the surface becoming rough. Especially when the volume of the added Au NBPs increased from 0.5 mL to 0.75 mL, the main peak red-shifted obviously from 950 nm to 980 nm. Combined with the morphological evolution of R-Au NBPs in Figure 1, the shape of R-Au NBPs evolved from rod-like to biconical. We infer that R-Au NBPs (with 0.75 mL Au NBPs) have the largest surface roughness, and their roughness decreases along with the further increase in Au NBP solution. As exhibited in Figure 2b, as well as the increase in Au NBP solution volume, the shape of R-Au NBPs evolved from being biconical to dumbbell-like, and the main peak red-shifted slightly, indicating an increase in the aspect ratio of R-Au NBPs [36]. Finally, when the volume of the Au NBPs increases to 2 mL, due to the reduced growth of Au at the ends of R-Au NBPs, the level of roughness is lowest, resulting in the main peak exhibiting a blue-shift.

### 3.2. Growth of R-Au/Pt-CdS Nanohybrids and Their Optical Properties

**Figure 3:**
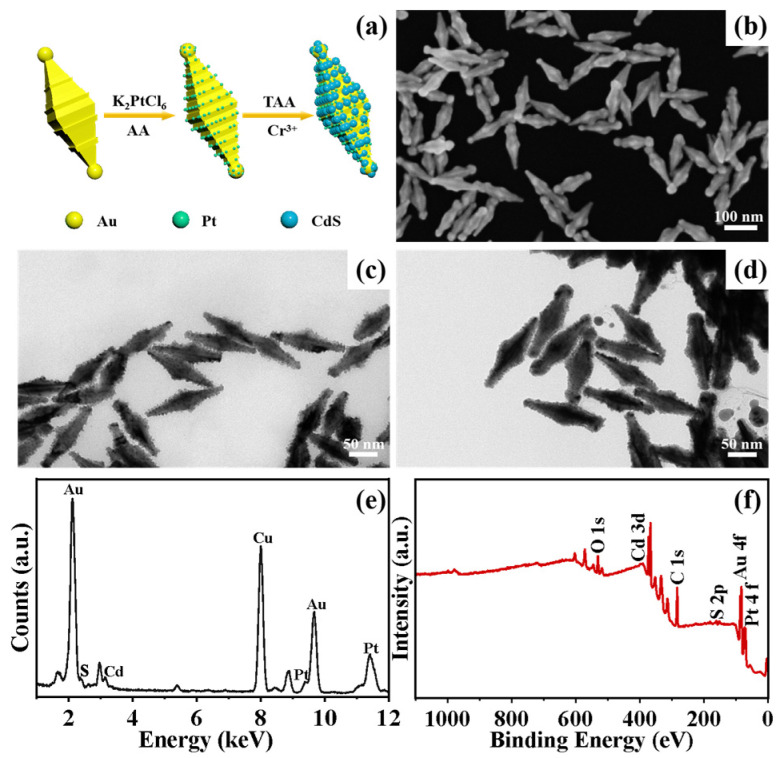
(**a**) Schematical illustration of the preparation of R-Au/Pt-CdS nanostructures. SEM image of R-Au NBPs (**b**) and TEM image of R-Au NBPs/Pt (**c**) and R-Au/Pt-CdS nanostructures (**d**). EDS pattern (**e**) and XPS survey (**f**) of R-Au/Pt-CdS nanostructures.

The detailed approach for the synthesis of R-Au/Pt-CdS nanohybrids is exhibited in Figure 3a. Initially, R-Au NBPs with a maximum surface roughness based on 0.75 mL Au NBPs were prepared and redispersed in 0.05 M hexadecyltrimethylammonium bromide (CTAB) solution, which were then used as templates (Figure 3b). Due to the curvature effect, the surfactant is more distributed on the smooth regions of R-Au NBPs than on its protrusions. Meanwhile, the ends and side surface protrusions of R-Au NBPs are exposed to the growth solution; then, Pt nanoparticles tend to decorate the surface of R-Au NBPs, as shown in Figure 3c. Due to Au and Ag sharing the same crystalline phase and having similar lattice parameters, in this approach, the Ag layer is used as a “bridge” to connect Au and Pt [25]. After being decorated with Pt, the average longitudinal length and average transverse length of the obtained R-Au/Pt nanostructures are 136.3 ± 2 nm and 46.4 ± 2 nm, respectively. There is only a slight increase in size compared to the R-AuNBPs in Figure 1c. On the one hand, this is because the amount of deposited Pt is very small. On the other hand, during the long Pt, the R-Au NBPs were subjected to minor etching. Finally, CTAB, AA, and thioacetamide (TAA) are added to the solution of R-Au/Pt in sequence. CdS nanoparticles are deposited on R-Au/Pt by controlling the amount of cadmium acetate. Since the lattice mismatch between Pt and CdS is larger than that between Au and CdS [37], the CdS tends to be preferentially deposited on the remaining surface of Au rather than Pt. The average transverse length of R-Au/Pt-CdS extends to 46.56 ± 2 nm, as shown in Figure 3d. The R-Au/Pt-CdS sample was dropped on a copper grid for energy-dispersive X-ray spectroscopic (EDS) analysis (Figure 3e). Spectral analysis indicates the presence of Au, Pt, S, and Cd within the R-Au/Pt-CdS nanohybrids. Meanwhile, the X-ray photoelectron spectroscopy (XPS) measurement was carried out to help analyze the composition and chemical state of R-Au/Pt-CdS nanohybrids. The XPS survey presented in Figure 3f verifies the presence of Au, Pt, Cd, and S in the nanohybrids.

**Figure 4:**
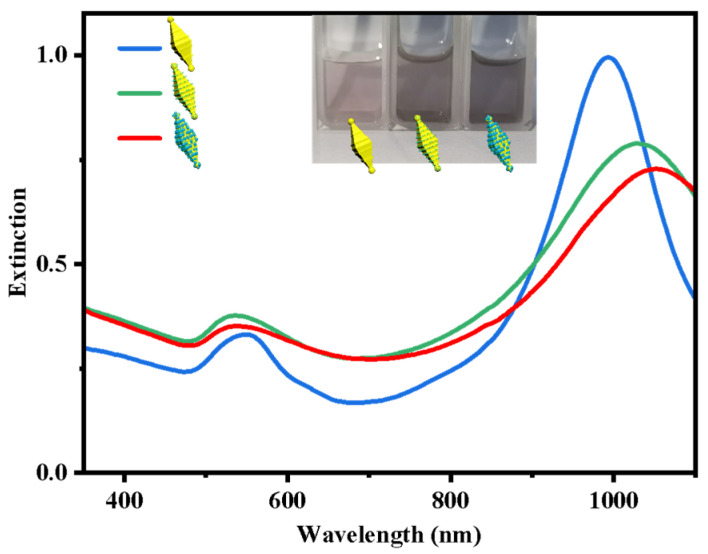
Extinction spectra of R-Au NBPs, R-Au NBPs/Pt, and R-Au/Pt-CdS nanostructures.

The plasmonic properties of these structure-adjustable nanostructures are investigated experimentally. The evolution of the experimental extinction spectra for different nanostructures is depicted in Figure 4. As previously described, the initial R-Au NBPs exhibit a transverse plasmon resonance peak at 548 nm and a longitudinal plasmon resonance peak near 994 nm. After the deposition of Pt particles on both tips and side surface protrusions of R-Au NBPs, the main peak red-shifts to around 1026 nm due to the increase in longitudinal size and the change in dielectric constant [38,39]. The surface plasmon resonance (SPR) intensity of the main peak is also reduced by the damping effect of Pt, accompanied by the broadened spectral width. Subsequently, the growth of CdS particles further red-shifts the main peak to approximately 1058 nm, similarly accompanied by a broadening of the spectral width due to the increase in the surrounding dielectric constant caused by CdS.

### 3.3. Photothermal Conversion Performance

**Figure 5:**
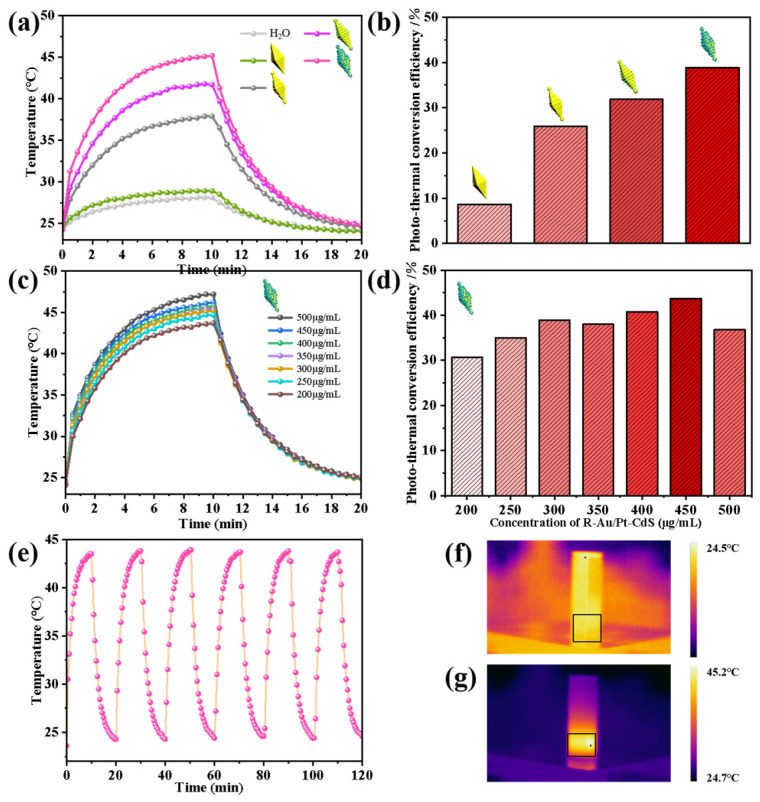
Photothermal temperature curves with 1064 nm laser irradiation and power of 1 w/cm^2^ (**a**); average photothermal conversion efficiencies of Au NBPs, R-Au NBPs, R-Au NBPs/Pt, and R-Au/Pt-CdS (**b**). Photothermal temperature curves (**c**) and average photothermal conversion efficiency (**d**) of R-Au/Pt-CdS with different concentrations. (**e**) Photothermal cycling test of R-Au/Pt-CdS (250 μg/mL). Relative near-infrared thermal images of R-Au/Pt-CdS nanohybrids before (**f**) and after (**g**) light irradiation for 10 min.

The photothermal conversion properties of these different samples were investigated by monitoring the temperature every 30 s under 1064 nm laser irradiation. The power of the laser was 1 w/cm^2^. All the samples kept the same molar concentration during the whole measurement. Figure 5a illustrates the temperature variation curve of different samples, including H_2_O, pure Au NBPs, R-Au NBPs, R-Au NBPs/Pt, and R-Au/Pt-CdS. After 10 min of irradiation, the temperature of H_2_O increased by only Δ*T* = 3.9 °C. The temperature increments Δ*T* for Au NBPs, R-Au NBPs, R-Au NBPs/Pt, and R-Au/Pt-CdS increased by 4.5, 13.6, 17.4, and 20.7 °C, respectively. Among these, R-Au/Pt-CdS nanohybrids exhibit the superior photothermal performance; the Δ*T* is 4.6 times greater than that of the pure Au NBPs. We calculate the photothermal conversion efficiency of each sample under the same experimental conditions (as shown in Figure 5b). The photothermal conversion efficiency of R-Au NBPs increased to 25.89%, which significantly surpassed the 8.65% efficiency of Au NBPs with a smooth surface. We believe that the enhancement of photothermal conversion efficiency of R-Au NBPs can be attributed to the larger specific surface area, the greater number of hotspots due to the rough surface, and stronger electric field intensity [19,40]. After being decorated with Pt nanoparticles, the efficiency of the R-Au NBPs/Pt reached 31.84%. This enhancement can be attributed to the improved damping effect induced by the Pt nanoparticles, which increased the availability of the active sites for catalytic reactions, leading to the enhancement of the photothermal conversion performance. Under identical test conditions, the R-Au/Pt-CdS nanohybrids displayed the best photothermal conversion efficiency of 38.88%, which is 4.49, 1.5, and 1.22 times higher than that of Au NBPs, R-Au NBPs, and R-Au NBPs/Pt, respectively. These results indicate that the CdS nanoparticles facilitate the generation of electron-hole pairs and further provide a new pathway for electron transformation in R-Au/Pt-CdS nanohybrids, which is the same result as previously reported [25]. To systematically illustrate the photothermal conversion performance of R-Au/Pt-CdS nanohybrids, the photothermal temperature variation curve of R-Au/Pt-CdS with different concentrations and photothermal cycling tests was measured and is shown in Figure 5c,e. In Figure 5c, as the concentration of R-Au/Pt-CdS increases, the temperature rises more rapidly. At a maximum concentration of 500 μg/mL, the temperature reaches 23.1 °C within 10 min under irradiation. However, as shown in Figure 5d, the photothermal conversion efficiency reaches a maximum at 43.73% when the sample concentration is 450 μg/mL. After a further increase in the concentration, the photothermal conversion efficiency instead decreased to 36.82%. We speculate that when the concentration of the test solution is too high, the nanoparticles may be more prone to aggregating, leading to a change in the absorption and scattering of light and reducing the photothermal conversion efficiency in contrast. In addition, Figure 5e illustrates the photothermal stability of R-Au/Pt-CdS nanohybrids. No noticeable decrease is observed in the temperature elevation. The R-Au/Pt-CdS nanohybrids show excellent photothermal stability, and the photothermal conversion efficiency is well maintained after six cycles. Figure 5f,g show the infrared thermal images of the aqueous solution of R-Au/Pt-CdS nanohybrids before and after light irradiation for 10 min. It can be seen from the image that after light irradiation, the temperature of the solution increased, while the volume of the solution decreased. It is expected that such three-component nanohybrids with rough Au cores and decorated with Pt and CdS are a promising candidate in many photocatalytic applications.

## 4. Conclusions

In conclusion, we employed a regrowth approach to modify the surface structure of Au nanobipyramids and obtained R-Au NBPs with tunable surface roughness. Subsequently, the growth of Pt protrusions on the surface of R-Au NBPs due to the curvature effect and the deposition of CdS on R-Au NBPs caused by lattice mismatch is achieved to construct R-Au/Pt-CdS nanohybrids. Through the analysis of their structure, morphology, and optical properties, we believe that rich hotspots and strong electric field intensity on the surface of rough Au cores, the damping effect and abundant active sites caused by Pt nanoparticles, and new electron transfer pathways originating from CdS deposition collectively enhance the final photothermal conversion performance of R-Au/Pt-CdS. Under near-infrared (1064 nm) irradiation, the outstanding photothermal conversion efficiency of R-Au/Pt-CdS is obtained. The optimized nanostructures have the highest photothermal conversion efficiency of 38.88%, which is 4.49 times higher than that of pure Au NBPs. Our research provides a potential strategy for constructing multicomponent hetero-nanostructures and optimizing the plasmon properties of metal nanocrystals.

## Data Availability

Data are contained within the article.
